# Extra-Medullary Hematopoiesis in Sickle Cell Disease Presenting as a Right Adrenal Mass

**DOI:** 10.7759/cureus.21334

**Published:** 2022-01-17

**Authors:** Folasade Ajayi, Meenakshi S Nali, Ruhma Ali, Aditya Patel, Hamid Shaaban

**Affiliations:** 1 Internal Medicine, Saint Michael's Medical Center, Newark, USA; 2 Hematology/Oncology, Saint Michael's Medical Center, Newark, USA

**Keywords:** mri images, adrenal mass, sickle cell anemia, extramedullary hematopoiesis, adrenal

## Abstract

Extramedullary hematopoiesis can occur during normal fetal development or as a compensatory mechanism in chronic anemia when the primary sites of hematopoiesis fail. When it occurs, it is mostly seen in sites such as the liver, spleen, and lymph nodes. Extramedullary hematopoiesis is seen in patients with abnormal morphology of red blood cells like thalassemia and abnormal red blood cell shape like hereditary spherocytosis. The incidence of extramedullary hematopoiesis in sickle cell disease is very rare. We report a case of focal adrenal extramedullary hematopoiesis in a 21-year-old patient with sickle cell disease who presented with pain in the right thorax. A retroperitoneal mass was seen in the MRI of the abdomen of the patient suggestive of extramedullary hematopoiesis. Our case highlights the importance of physician awareness of this rare pathology for proper diagnosis and management.

## Introduction

Extramedullary hematopoiesis is defined as the production of erythroid and myeloid progenitor cells in organs outside the bone marrow. It occurs in diverse conditions, including fetal development, normal immune responses, and pathological circumstances. During fetal development, before the formation of mature marrow, extramedullary hematopoiesis occurs in the yolk sac, fetal liver, and spleen. Most frequently, this response occurs in the spleen and liver for the production of antigen-presenting and phagocytes. Extramedullary hematopoiesis (EMH) can also occur in pathological conditions when bone marrow is inhabitable for stem and progenitor cells, including myelofibrosis, lymphoma, leukemia, or with marrow hyperactivity in conditions such as congenital hemolytic anemia like thalassemia and spherocytic anemia. Common known sites for extramedullary hematopoiesis are the liver, spleen, lymph nodes, and mediastinum. We report the first case of focal retroperitoneal EMH in sickle cell anemia depicted on MRI.

## Case presentation

A 21-year-old African American male with a past medical history of homozygous sickle cell disease presented to the emergency department with a complaint of pain over the right thorax for the last two days. Patient described his pain as a clawing sensation that is worse on inspiration and movement. Patient has had frequent hospitalizations for sickle cell crisis. He denied any headache, shortness of breath, palpitations, or abdominal pain. He takes folic acid regularly. On admission, the patient was afebrile, normotensive, and saturating 96% on room air. Physical examination including cardiac and respiratory examination was normal. Initial laboratory workup revealed an elevated reticulocyte count, total bilirubin, and lactate dehydrogenase (LDH). The initial laboratory values are shown in Table 1.

**Table 1:** Initial laboratory values.

Laboratory Parameters	Values	Reference Range
Sodium	138	136-145 mmol/L
Potassium	4.3	3.5-5.3 mmol/L
Chloride	110	98-110 mmol/L
Blood urea nitrogen (BUN)	7	6-24 mg/dL
Creatinine	0.7	0.6-1.2 mg/dL
Aspartate transaminase (AST)	33	10-36 U/L
Alanine transaminase (ALT)	27	9-46 U/L
White blood cell (WBC)	9.4	4.4-11 x 10^3^/uL
Hemoglobin	7.8	13.5-17.5 g/dL
Platelets	311	150-450 x 10^3^/uL
Lactate dehydrogenase (LDH)	874	122-222 U/L
Reticulocyte count	7.8	0.5-1.5%
Total bilirubin	2	0.2-1.2 mg/dL

**Figure 1:**
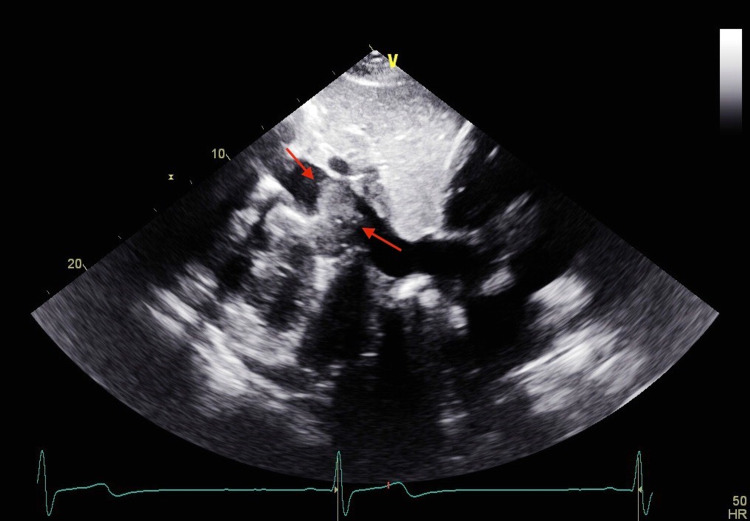
Cystic mass of 2.8 x 3.2 cm compressing the inferior vena cava on ECHO. ECHO: echocardiogram

**Figure 2:**
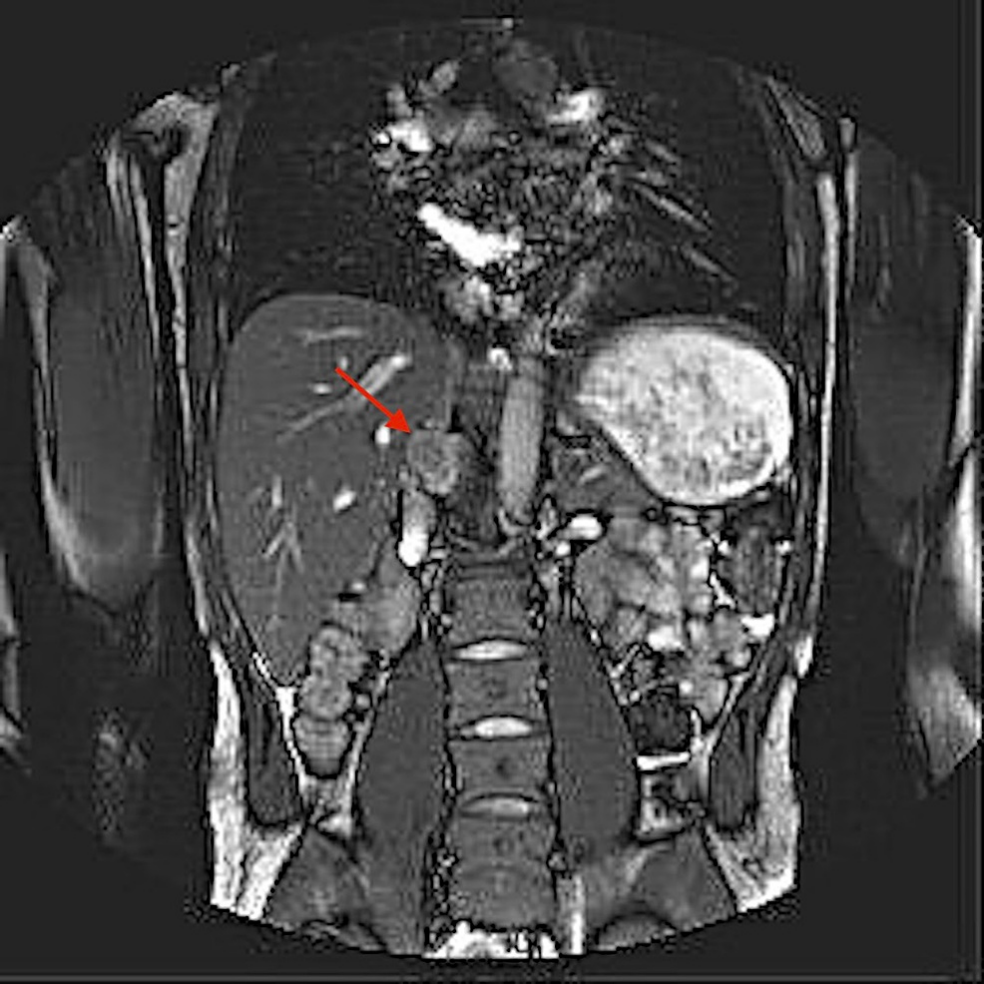
Progressively enhancing mass of 4.7 x 2.7 cm on MRI of the abdomen.

Peripheral blood smear showed few giant cells, anisocytosis, and sickle cells. Chest x-ray revealed no evidence of cardiopulmonary disease. Echocardiogram showed a 2.8 x 3.2 cm cystic mass compressing the inferior vena cava with overlying fibrinous material (Figure 1). Ultrasound of the abdomen showed a mass impressing on the posterior wall of the suprarenal inferior vena cava extending into the lumen. Magnetic resonance imaging (MRI) of the abdomen with and without contrast revealed a 4.7 x 2.7 cm progressively enhancing right retroperitoneal mass (Figure 2).

The patient was started on symptomatic management and close imaging surveillance for the mass was recommended. Follow-up after two months in the outpatient hematology clinic showed a stable mass with no symptoms.

## Discussion

Sickle cell disease is a common, congenital blood disorder that is characterized by abnormally shaped red blood cells [1]. This deformation impairs the ability of the cell to pass through the small vascular channels resulting in congestion of the vascular beds. These sickle-shaped erythrocytes then undergo intravascular hemolysis and are destroyed at an increased rate leading to chronic anemia [2]. In these circumstances, extramedullary production of erythrocytes can be initiated to supplement the increased destruction of red blood cells [3].

Extramedullary hematopoiesis (EMH) is a reactive mechanism that can be seen in congenital hemolytic anemias. EMH can be divided into two main groups. The first group is seen in hemolytic disorders where the marrow shows tremendous activity and paraosseous foci which may result from herniation of the medullary tissue from the underlying bone. The second group shows extraosseous foci and is seen when marrow activity is ineffective [4]. Common sites of EMH include the reticuloendothelial system such as spleen, lymph node, and mediastinum; however, the process can include virtually any tissue of the body [5]. Cases have been reported of EMH in the bowel, breast, pleura, and brain [6]. In our literature review, we found cases of retroperitoneal EMH after thalassemia and hereditary spherocytosis [7]. EMH presenting as retroperitoneal mass in a patient with sickle cell disease has rarely been presented. We report a case of EMH found as a retroperitoneal mass in a patient with sickle cell disease. Our patient presented with pain in the right thorax with MRI abdomen findings suggestive of an adrenal retroperitoneal mass. Patient was also found to have periportal and left retroperitoneal lymph nodes on diffusion-weighted imaging. Patient was normotensive with a normal metanephrine level. This incidental adrenal mass along with lymph node proliferation in the retroperitoneum is highly suggestive of EMH.

The prevalence of adrenal incidentaloma is about 0.5-2% on the abdominal computed tomography (CT) scan in the general population [8]. EMH is a rare cause of incidentaloma but may be seen in patients with hematological disorders like thalassemia. The exact mechanism of EMH is unknown but several hypotheses have been suggested. It is suggested that since adrenal glands have hematopoietic capacity during the fetal period, anomalous activation of these embryonic stem cells can occur during the disease state. Another hypothesis suggests that embolization of the hematopoietic stem cells and homing in the adrenal gland may play a role in this phenomenon. Chronic hypoxia has also been reported as a presumptive cause of EMH [9]. EMH is normally asymptomatic and discovered as an incidental finding. Symptomatic cases can be seen due to the mass effect created by compression of the adjacent organs [10].

Diagnosis of EMH is based on the clinical picture, laboratory data, and the use of imaging modalities [4]. MRI is considered to be the diagnostic modality of choice and is superior to CT scan. MRI can effectively delineate the extent of the EMH mass and has no radiation exposure [11]. Biopsy is not always indicated but can be utilized for definitive diagnosis. Treatment modalities for patients with EMH depend on the location and symptoms. Surgery, local radiation, blood transfusion, and hydroxyurea are some treatment approaches. Surgical excision is indicated if the mass is more than 6 cm in diameter [9]. In our case, the size of the tumor was not large enough for excision. Hydroxyurea is used as an adjuvant therapy due to its myelosuppressive properties [12]. Hypertransfusion is another rising therapy that is postulated to reduce the risk of EMH by bone marrow suppression. In our patient imaging surveillance with close follow-up was advised since the patient was asymptomatic.

## Conclusions

This case suggests that EMH should be taken into consideration when retroperitoneal masses are identified in patients with blood dyscrasias. This case is unique due to the location of the EMH. The patient was followed closely by imaging surveillance to monitor the size of the mass. This case also suggests that increased awareness of this rare entity is needed to avoid aggressive therapeutic measures in asymptomatic patients. In such patients, conservative management techniques should be employed.

## References

[1] Saito N, Nadgir RN, Flower EN, Sakai O (2010). Clinical and radiologic manifestations of sickle cell disease in the head and neck. RadioGraphics.

[2] Delicou S (2017). Extramedullary haemopoiesis in hemoglobinopathies. J Hematol Transfus.

[3] Lonergan GJ, Cline DB, Abbondanzo SL (2001). Sickle cell anemia. RadioGraphics.

[4] Jelali MA, Luciani A, Kobeiter H (2006). MRI features of intrahepatic extramedullary haematopoiesis in sickle cell anaemia. Cancer Imaging.

[5] Tayari N, Ahrar MH, Jafarpishe MS (2013). Case report of the extramedullary hematopoiesis presented as a hypervascular intracranial mass. Adv Biomed Res.

[6] Clark CA, Worden CP, Thorp BD (2020). Extramedullary hematopoiesis in the sinonasal cavity: a case report and review of the literature. Allergy Rhinol (Providence).

[7] Hassanzadeh M (2013). Extramedullary hematopoiesis in thalassemia. N Engl J Med.

[8] Azarpira N, Esfahani MH, Paydar S (2014). Extramedullary hematopoiesis in adrenal gland. An uncommon cause of adrenal incidentaloma in sickle cell disease. Iran J Pediatr.

[9] Barzon L, Sonino N, Fallo F, Palu G, Boscaro M (2003). Prevalence and natural history of adrenal incidentalomas. Eur J Endocrinol.

[10] Kolev NH, Genov PP, Dunev VR, Stoykov BA (2020). A rare case of extramedullary hematopoiesis in adrenal mass. Urol Case Rep.

[11] Verani R, Olson J, Moake JL (1980). Intrathoracic extramedullary hematopoiesis: report of a case in a patient with sickle-cell disease-beta-thalassemia. Am J Clin Pathol.

[12] Wang A, Carberry N, Solli E, Gillick J, Islam H, Hillard V (2016). Spinal cord compression secondary to extramedullary hematopoiesis: case report and review of the literature. Case Rep Oncol.

